# Tetrahedral framework nucleic acids‐based delivery of microRNA‐155 alleviates intervertebral disc degeneration through targeting Bcl‐2/Bax apoptosis pathway

**DOI:** 10.1111/cpr.13689

**Published:** 2024-06-20

**Authors:** Zhuhai Li, Yuanlin Tang, Lihang Wang, Kai Wang, Shishu Huang, Yu Chen

**Affiliations:** ^1^ Department of Orthopedic Surgery and Orthopedic Research Institute West China Hospital, Sichuan University Chengdu China; ^2^ Department of Spine Surgery The People's Hospital of Guangxi Zhuang Autonomous Region Nanning China; ^3^ State Key Laboratory of Oral Diseases, National Clinical Research Centre for Oral Diseases, West China Hospital of Stomatology, Sichuan University Chengdu China; ^4^ Department of Spine Surgery Beijing Jishuitan Hospital Guizhou Hospital Guiyang China

## Abstract

Intervertebral disc degeneration (IDD) is one of the most common causes of chronic low back pain, which does great harm to patients' life quality. At present, the existing treatment options are mostly aimed at relieving symptoms, but the long‐term efficacy is not ideal. Tetrahedral framework nucleic acids (tFNAs) are regarded as a type of nanomaterial with excellent biosafety and prominent performance in anti‐apoptosis and anti‐inflammation. MicroRNA155 is a non‐coding RNA involved in various biological processes such as cell proliferation and apoptosis. In this study, a complex named TR155 was designed and synthesised with microRNA155 attached to the vertex of tFNAs, and its effects on the nucleus pulposus cells of intervertebral discs were evaluated both in vitro and in vivo. The experimental results showed that TR155 was able to alleviate the degeneration of intervertebral disc tissue and inhibit nucleus pulposus cell apoptosis via Bcl‐2/Bax pathway, indicating its potential to be a promising option for the treatment of IDD.

## Graphical abstract

Tetrahedral nucleic acids loaded with microRNA155 can alleviate intervertebral disc degeneration in rats and inhibit nucleus pulposus cell apoptosis via Bcl‐2/Bax apoptosis pathway.

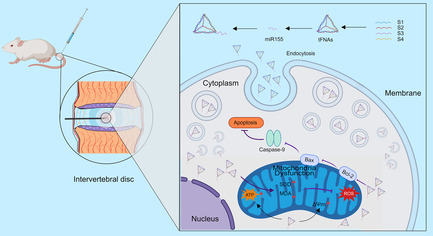

## 1 INTRODUCTION

Low back pain (LBP) is a major public health problem in the modern society and it is the leading cause for labour loss.^1^ An epidemiological study estimated that 266 million individuals worldwide are diagnosed with LBP annually.^2^ Intervertebral disc degeneration (IDD) is believed to be closely associated with the development of LBP.^3^ The intervertebral disc (IVD) is a complex structure, consisting of nucleus pulposus (NP) and annulus fibrosus (AF). Currently, a widely held consensus among scholars is that NP is considered the most critical component of the disc for maintaining the pressure gradient and ensuring the infiltration and diffusion of metabolic products and nutrients. Pathological alterations within NP are therefore thought to play a significant role in both initiating and exacerbating IDD.^4^ Excessive apoptosis of nucleus pulposus cells (NPCs) can lead to a decrease in cell density and reduction in extracellular matrix (ECM) secretion, eventually resulting in IDD.^5^ Due to this point, the inhibition of NPC apoptosis may help slow the disease progression of IDD.

There is evidence that mitochondrial pathway participates in regulating cell apoptotic process in IDD.^6^ Specifically, mitochondrial homeostasis imbalance results in excessive generation of reactive oxygen species (ROS), leading to lipid peroxidation, DNA damage and consequently, cell apoptosis.^7^ It has been reported that ROS is a potent inducer of apoptosis in human NPCs and is considered one of the core mediators in the occurrence and progression of IDD.^8^ During the IDD disease progression, mechanical loading, nutrition deprivation and pro‐inflammatory cytokines can all potentially lead to excessive ROS production, decrease mitochondrial membrane potential (MMP), and eventually result in mitochondrial dysfunction.^9^ MicroRNAs are a category of non‐coding RNAs that modulate the protein expression of target genes by establishing complementary pairs with the transcriptional mRNA sequences of these specific genes.^10^ As a member of the microRNA (miR) family, miR‐155 participates in various biological processes including cell proliferation and apoptosis.^11^, ^12^ The effectiveness of miR‐155 varies depending on the specific stimulus involved.^13^ Deregulated miR‐155 enhances Fas‐mediated apoptosis in human IDD by specifically targeting FADD and caspase‐3.^14^ Besides, miR‐155 can also inhibit matrix degradation and cell apoptosis induced by cholesterol via targeting RORα in NPCs.^12^ The above evidence suggests a potential involvement of miR‐155 as a promising treatment option for IDD. Nevertheless, the existence of nucleases within cells renders oligonucleotides susceptible to degradation and impedes their efficient cellular entry. Hence, a reliable delivery system is needed to facilitate the ingress of oligonucleotides into target cells.

DNA nanomaterials have received extensive attention across diverse fields, including biomedical therapy and gene delivery.^15^ Multiple studies indicate that tFNAs exhibit unparalleled advantages in anti‐inflammatory and anti‐apoptotic responses.^16^, ^17^, ^18^ A number of investigations have also revealed that tFNAs function as a proficient delivery system for small‐molecule drugs and microRNAs.^19^, ^20^, ^21^ Therefore, tFNAs can serve as an ideal vehicle for delivering microRNAs into cells to exert their corresponding biological effects. In our study, we utilised tFNAs for carrying miR‐155 and successfully constructed a complex calledTR155. We intervened NPCs with TR155 in vitro and observed its impact on the IDD mice model in vivo. Our findings demonstrated the effectiveness of TR155 in the inhibition of NPC apoptosis as well as the alleviation of IDD in mice, thus suggesting an alternative approach to address IDD‐related diseases in the future.

## 2 METHODS

### 2.1 Materials

Four single‐strand DNA (ssDNA) and microRNA‐155 mimics were procured from Sangon (Shanghai, China). The Cell Counting Kit‐8 (CCK‐8) was acquired from Abbkine Scientific Corp (Wuhan, China). The fluorescent 2′,7′‐dichlorofluorescein diacetate (DCFH‐DA) Kit was obtained from Beyotime (Shanghai, China). ProteoPrep® Total Extraction Sample Kits were sourced from Keygen (Jiangsu, China). Annexin V‐FITC Apoptosis Detection Kit, Calcein/PI live/dead viability/Cytotoxicity Assay Kit, ROS Assay Kit, Mitochondrial membrane potential assay kit with Rhodamine 123, Total Superoxide Dismutase Assay Kit with WST‐8, Lipid Peroxidation MDA Assay Kit and all antibodies utilised for protein determination were purchased from Beyotime (Shanghai, China).

### 2.2 Synthesis of tFNAs and tFNAs‐miR155 (TR155)

Initially, a pH 8.0 TM buffer, comprising 10 mM Tris–HCl and 50 mM MgCl2, was prepared. Subsequently, equimolar concentrations of the four single‐strand DNAs were introduced into the TM buffer. Following thorough mixing and centrifugation, the blended solution underwent a heating step at 95°C for 10 min, followed by a cooling period to 4°C for 20 min, resulting in the successful formation of tFNAs. At the same concentration, the modified microRNA‐155 mimics were subjected to the synthesised tFNAs, and the mixture was incubated at room temperature for 30 min to obtain TR155. The base sequence of the four ssDNAs and modified miRNA‐155 mimics are detailed in Table 1.

**TABLE 1:** Base sequence of each ssDNA (capital letters denote DNA monomers and lowercase letters denote RNA monomers).

ssDNA	Base sequence (from 5′ to 3′)
S1	ATTTATCACCCGCCATAGTAGACGTATCACCAGGCAGTTGAGACGAACATTCCTAAGTCTGAA
S2	ACATGCGAGGGTCCAATACCGACGATTACAGCTTGCTACACGATTCAGACTTAGGAATGTTCG
miR155‐S3	uuaaugcuaaucgugauaggggTTACTACTATGGCGGGTGATAAAACGTGTAGCAAGCTGTAATCGACGGGAAGAGCATGCCCATCC
S4	ACGGTATTGGACCCTCGCATGACTCAACTGCCTGGTGATACGAGGATGGGCATGCTCTTCCCG

### 2.3 Characterisation of tFNAs and TR155

In accordance with established research procedures,^22^ we utilised polyacrylamide gel electrophoresis (PAGE) and dynamic light scattering (DLS) to identify disparities in the molecular weights of tFNAs and TR155. We employed a nanoparticle size analyser to identify discrepancies in the zeta potential and particle sizes of the two reagents, aiming to assess the success of the synthesis.

### 2.4 Isolation and culture of rat NPCs

Nucleus pulposus cells were extracted from the nucleus pulposus tissue of 4‐week‐old Sprague–Dawley (SD) rats. In brief, the NP tissue from each caudal vertebra was aseptically dissected and subsequently immersed in type II collagenase (0.25%) for a duration of 2 h at 37°C. Following filtration, the supernatant was centrifuged at 1000 *g* for 5 min. After PBS washes, the initial culture was conducted utilising MEMα with 10% FBS in a humidified incubation chamber at 37°C with 5% CO_2_. The solution was refreshed every 72 h, and passaging occurred upon reaching 80% cell density. In subsequent experiments, cells from the third passage were employed and a concentration of 100 ng/mL TNF‐α was added to the culture medium for 24 h to simulate a degenerated inflammatory environment. The working concentrations for microRNA155 mimics, tFNAs and TR155 were uniformly established at 1 nM.

### 2.5 Cellular uptake of TR155

To examine the cellular absorption of TR155, S3 was marked with Cy5. After adhering to the synthesis procedure outlined above, we obtained Cy5‐tFNAs‐miR‐155, which is observable through a confocal laser microscope (CLSM) (TCS SP8, Leica). We seeded NPCs in a 24‐well plate containing MEMα for 24 h. Subsequently, we replaced the original cell culture medium with a culture medium containing Cy5‐tFNAs‐miR‐155 and incubated for an additional 24 h. NPCs were washed with PBS (HyClone) and subsequently fixed with a 4% cold polyoxymethylene solution for 15 min. Following another round of rinsing, cells were stained with phalloidin and DAPI for 10 min each. Ultimately, we examined cellular uptake using a CLSM and captured the images.

### 2.6 Assessment of cell viability

We seeded NPCs in a 96‐well plate with 6000 cells per well, followed by sequential pre‐incubation in 10% FBS MEMα cell culture medium for 24 h. Then, the cells were divided into five groups: a blank group and TR155 treatment groups with concentrations of 62.5, 125, 250 and 375 nM, respectively. The medium was incubated for an additional 24 h. After three PBS washes, 10 μL of the CCK‐8 solution was added to each well. Subsequently, the 96‐well plate underwent a 2‐h incubation period, following which it was inserted into a microplate reader to quantify the absorbance at 450 nm.

### 2.7 Apoptosis assay

#### 2.7.1 Flow cytometry

The NPCs were seeded in a 6‐well plate and divided into five groups: the blank group, TNF‐α group, miR155 group, tFNAs group and TR155 group. After treating each cell group with the respective drugs and incubating for 24 h, the cells were washed with PBS, collected, and resuspended in Annexin V Binding Buffer. Subsequently, Annexin V‐FITC reagent and PI reagent were added to the cell suspension, followed by a 15‐min incubation at room temperature. Finally, the samples were analysed using a flow cytometer.

#### 2.7.2 Live–dead staining

To evaluate cellular viability, a live–dead staining procedure was conducted. Cells were marked with calcein AM, representing live cells in green, and propidium iodide (PI), indicating dead cells in red. The NPCs were cultured in a 24‐well plate and categorised into five groups: the control group, TNF‐α‐treated group, miR155‐treated group, tFNAs‐treated group and TR155‐treated group. Following the treatment of each cell group with the designated drugs and a 24‐h incubation period, the cells underwent a PBS wash. Subsequently, 250 μL of Calcein AM/PI detection solution was added to each well and incubated at 37°C in a humidified atmosphere with 5% carbon dioxide for 30 min. Finally, the samples were observed under a fluorescence microscope (Ti2, Nikon, Japan). Cell viability (%) was determined by tallying the number of living and deceased cells within the 3D structures generated through LAS X Image Analysis (3D) software, employing the subsequent equation:

$$\text{Cell viability}\left(\%\right)=\begin{array}{l} \text{Live cells(green)}/\text{Total number of cells}\left(\text{green}+\mathrm{red}\right)\\ {}*100\%. \end{array}$$

The quantification results were expressed as the mean ± standard deviation (SD) from two independent experiments, each consisting of two replicates and three images per replicate.

### 2.8 Quantitative polymerase chain reaction

The experimental cell RNA was isolated utilising the RNeasy Kit (Yeasen Biotechnology, Shanghai, China), and the quantification of the RNA samples was conducted using a spectrophotometer. Following quantification, cDNA synthesis kits (Yeasen Biotechnology, Shanghai, China) were employed to perform reverse transcription on each total RNA sample. Subsequently, the evaluation of target mRNA expression was conducted through Quantitative polymerase chain reaction (qPCR) using SYBR Green Master Mix (Yeasen Biotechnology, Shanghai, China) and an.

Applied Biosystems SimpliAmp PCR (Thermo Fisher Scientific, MA, USA). GAPDH served as the reference gene in the experiment.

### 2.9 Immunofluorescence staining

Nucleus pulposus cells were seeded into a 48‐well plate and incubated for 24 h. Subsequently, the culture medium was replaced and the corresponding drugs were added according to the aforementioned group. The cells were further incubated for an additional 24 h. Following PBS washing, the cells were fixed with 4% paraformaldehyde for 15 min, permeabilised with 0.5% Triton X‐100 for 10 min. The samples were then incubated with 5% goat serum for 1 h, followed by the addition of diluted primary antibodies and overnight incubation in a 4°C refrigerator. The concentration of the primary antibodies were as follows: anti‐Col II (Affinity, AF0135, 1:200), anti‐BMP2 (HUABIO, ER80602, 1:200), anti‐LC3B (Immunoway, YN5524, 1:200), anti‐SQSTM1/P62 (HUABIO, R1309‐8, 1:200), anti‐Bcl‐2 (Affinity, AF6139, 1:200), anti‐Bax (Affinity, AF0120, 1:200) and anti‐Caspase9 (Affinity, AF6348, 1:200). Afterwards, we performed three washes and then treated the samples with a secondary antibody (Affinity, S0006, 1:500) at 37°C for 1 h. Subsequently, DAPI and phalloidin were applied to stain the nucleus and cytoskeleton, respectively. The samples were examined using a fluorescence microscope (Nikon Ti2, Japan).

### 2.10 Detection of mitochondrial function

#### 2.10.1 Reactive oxygen species assay

We utilised the ROS Fluorometric Assay Kit (E‐BC‐K138‐F, Elabscience Biotechnology) to assess intracellular ROS. All procedures were conducted according to the manufacturer's instructions. Briefly, after washing NP cells three times with Buffer Solution, they were stained with DCFH‐DA in the dark at 37°C for 30 min. Subsequently, the NP cells were washed three additional times with Buffer Solution to reduce interference from excess DCFH‐DA. Utilise a fluorescence microscope (Nikon Ti2, Japan) to assess the fluorescence intensity of cells in each group.

#### 2.10.2 Measurement of mitochondrial membrane potential

The assessment of the MMP was conducted utilising the Rhodamine 123 Assay Kit (C2008S, Beyotime) following the guidelines provided by the manufacturer. In summary, cells were initially rinsed with PBS, exposed to 1 mL of the rhodamine 123 staining working solution, and then incubated at 37°C for 30 min within a cell incubator. Subsequently, the cells were subjected to two washes using fresh pre‐warmed complete medium at 37°C before being examined under a fluorescence microscope (Nikon Ti2, Japan).

#### 2.10.3 ATP production assay

To quantitate intracellular ATP production, this study utilised an ATP bioluminescence assay kit (S0026; Beyotime). NPCs were homogenised, followed by centrifugation. The resulting supernatants were then combined with the ATP detection working solution in a white 96‐well plate. The samples were analysed using a chemiluminescence analyser (ChemiDoc MP Imaging System, Bio‐Rad), and after plotting the standard curve, the ATP concentration of each group's samples was calculated.

#### 2.10.4 Detection of superoxide dismutase and malondialdehyde

The superoxide dismutase (SOD) activity and malondialdehyde (MDA) content in the samples were assessed utilising kits obtained from Beyotime Biotech, in accordance with the manufacturer's guidelines.

### 2.11 RNA‐Seq and bioinformation analysis

Library preparation and RNA sequencing were conducted utilising an Illumina HiSeq/Illumina NovaSeq/MGI2000 instrument at Azenta Life Sciences (Suzhou, China), using RNA extracted from cultured cells. Hisat2 (v2.0.1) was used to index reference genome sequence and clean data were aligned to reference genome via software Hisat2 (v2.0.1). In the beginning transcripts in fasta format are converted from known gff annotation file and indexed properly. Then, with the file as a reference gene file, HTSeq (v0.6.1) estimated gene and isoform expression levels from the pair‐end clean data. Differential expression analysis used the DESeq2 Bioconductor package. To investigate the functional mechanism of TR155, genes exhibiting a fold change in expression greater than 2 or less than 0.5, with a corresponding *p*‐value less than 0.05, were categorised as up‐regulated or down‐regulated, respectively. GOSeq(v1.34.1) was used identifying gene ontology (GO) terms that annotate a list of enriched genes with a significant *p*_adj_ less than 0.05. Gene Ontology and Kyoto Encyclopaedia of Genes and Genomes (KEGG) pathway analyses were conducted, and pathways with a *p*‐value below 0.05 were considered statistically significant.

### 2.12 Western blot

The NPCs underwent a rinsing process, were collected, and subsequently lysed using a lysis buffer. Subsequently, a BCA assay was performed to quantify the protein concentration. To denature the proteins, the solution underwent boiling in SDS buffer, following which the proteins were separated using SDS‐PAGE. Upon transferring the proteins onto PVDF membranes, a blocking step was executed by incubating them with skim milk for a duration of 30 min. Afterward, the membranes underwent incubation with the primary antibody, followed by the respective secondary antibody. Ultimately, the membranes were exposed using an enhanced chemiluminescence detection system (Bio‐Rad). The ImageJ v1.52a software was employed for the analysis of the grey value of each membrane. β‐Actin functioned as the internal control for adjusting the sample loading quantities.

### 2.13 Establishment of rat IDD models and therapeutic intervention

Animal experiments were approved by the Animal Ethics Committee, West China Hospital, Sichuan University. Male SD rats weighing 250–300 g were included in this study and divided into six groups: control group, Sham group, PBS group, miR155 group, tFNAs group, and TR155 group. They were anaesthetised by continuous inhalation of isoflurane using an animal anaesthesia apparatus. Subsequently, 6–9 IVDs were punctured sequentially at the centre of the discs using 23G needles, rotated for 5 s, and maintained for 30 s. Subsequently, 20 μL of the respective drugs were injected into each intervertebral disc through a 33G needle based on the grouping, excluding the negative control group and Sham group.

### 2.14 Histological assessment

Following euthanasia, the IVDs were extracted from each rat and immersed in 10% formalin for 1 week. Subsequently, they underwent a 6‐week decalcification process in 10% EDTA solution and were later embedded in paraffin blocks. The samples were sectioned into 5 μm‐thick histological slices encompassing the end plates, AF and NP. These sections were then stained with either H & E or Safranin‐O/Fast Green. The histological grading was assessed based on the methodology established in prior studies.^23^ In order to assess the expression of aggrecan and MMP‐13 in the tissues, immunohistochemical staining for aggrecan and MMP‐13 was performed on the sections. Subsequent semi‐quantitative analysis of the immunohistochemical staining was carried out using Image J software.

### 2.15 Imaging evaluation

After an 8‐week post‐surgery period, three rats were randomly chosen from each group for X‐ray and MRI evaluations conducted prior to their sacrifice. The assessment of the disc height index (DHI) for the experimental discs followed established protocols derived from prior research.^24^ Employing the Pfirrmann classification method,^25^ MRI images were classified into grades I to V through the evaluation of T2‐weighted signal intensity.

### 2.16 Statistical analysis

The data are reported as means ± SD. Statistical analysis, conducted using GraphPad Software (USA), employed a one‐way ANOVA alongside a Tukey's multiple comparison test to assess variations among groups. Statistical significance was established for *p*‐values less than 0.05.

## 3 RESULTS

### 3.1 Synthesis and characterisation of tFNAs and TR155

**FIGURE 1:**
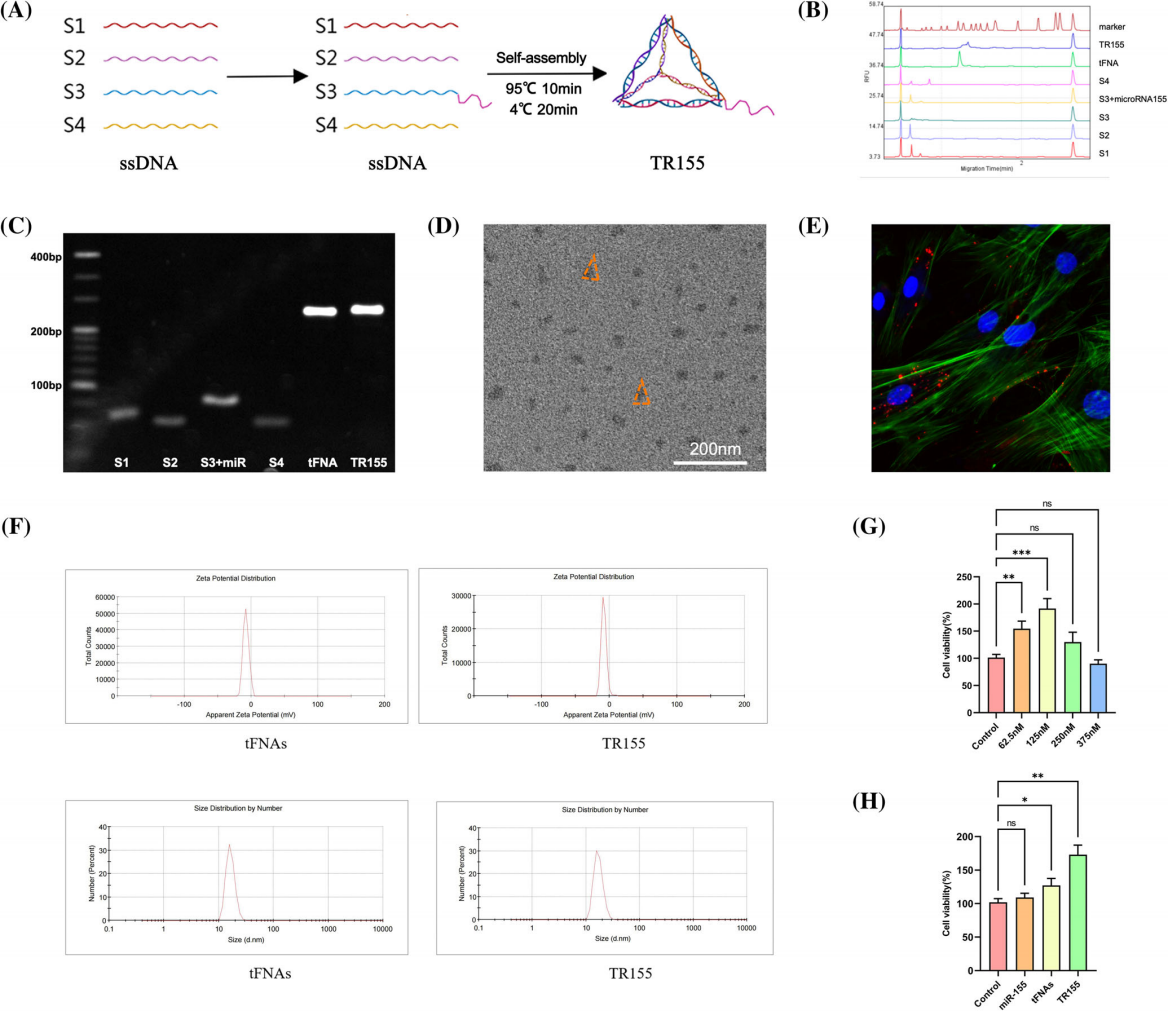
Synthesis, characterisation and cytotoxicity test of the TR155. (A) Synthesis illustration of TR155. (B) High‐performance capillary electrophoresis results of high‐performance capillary electrophoresis results of tFNAs. (C) The results of polyacrylamide gel electrophoresis (PAGE). (D) CLSM images of TR155. Scale bar: 200 nm. (E) Immunofluorescence images of internalised TR155 by NPCs (cytoskeleton: green, nucleus: blue, TR155: red). (F) Particle size and potential of tFNA and TR155. (G) The cytotoxicity of TR155 to NPC was detected by CCK8. (H) Effects of TR155, tFNA and miR‐155 at the same concentration on proliferation of NPCs. Data represented the mean ± SD. Statistical analysis was performed with one‐way ANOVA. (*) *p* < 0.05; (**) *p* < 0.01; (***) *p* < 0.001. CLSM, confocal laser microscope; NPC, nucleus pulposus cell; tFNA, tetrahedral framework nucleic acid.

The schematic diagram in Figure 1A describes the synthesis process of tFNAs and TR155. Four single strands (S1–S4) were self‐assembled into a tetrahedron via base‐pairing principles. In these single strands, the sticky end designed on the S3 strand is complementary to the sticky end on microRNA‐155. After forming a tetrahedral structure, the microRNA connects to one of the vertices of the tFNAs, forming TR155. The synthesis of tFNA was detected by PAGE (Figure 1C), and the results showed that the molecular weight of the single‐strand DNA was significantly different from that of tFNA, which was consistent with the theoretical value. In addition, the molecular weight of TR155 was larger than that of tFNA, indicating that microRNA‐155 had been successfully connected to tFNA. The synthesis of tFNAs was detected by capillary electrophoresis, and the result (Figure 1B) was consistent with the PAGE image. The shape of tFNA was observed by transmission electron microscopy, which demonstrated a triangular structure and a diameter between 10 and 20 nm (Figure 1D). According to the size and zeta potential distribution analysis, the surface negative charge of tFNAs was about −7.88 mV, and the surface charge of TR155 was about −8.23 mV when microRNA155 was loaded. The particle size of tFNAs was about 10 nm, and that of TR155 was about 13 nm (Figure 1F). To detect the cellular uptake of TR155, we labelled S3 with Cy5 and observed the endocytosis of TR155 through the CLSM (Figure 1E), which showed that TR155 successfully entered the cell.

### 3.2 Effect of TR155 on NPCs

Previous studies have shown that tFNAs exhibited a non‐toxic nature towards cells and demonstrated a pronounced capability in promoting cell proliferation.^19^, ^26^ In this study, we first evaluated the cytotoxicity of tFNAs, miR‐155 and TR155 on NPCs, respectively. Using the Cell Counting Kit‐8 (CCK‐8), we examined TR155's impact at different concentrations (62.5, 125, 250 and 375 nM) on cell proliferation. Notably, after 24 h, 125 nM of TR155 showed a significant proliferative effect on NPCs (Figure 1G). Hence, 125 nM was selected as the optimal concentration. Simultaneously, experiments with miR‐155 and tFNAs at an equivalent concentration served as control groups to assess miR‐155's effects without vector assistance. As shown in the Figure 1H, miR‐155 did not demonstrate a significant proliferative effect on NPCs, while tFNAs promoted the proliferation of NPCs. To delve into specifics, TR155 demonstrated a more pronounced promoting effect on the vitality of NPCs.

**FIGURE 2:**
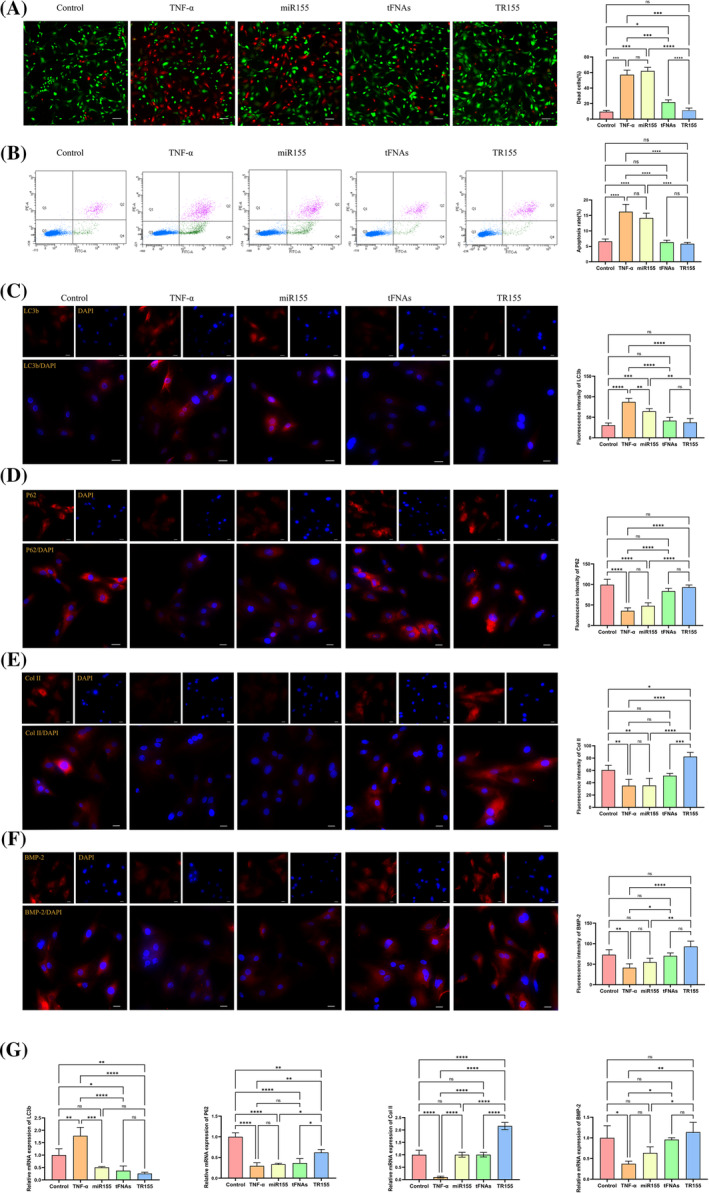
Effect of TR155 on NPCs. (A) Live‐dead staining to evaluate cellular viability of NPCs proliferation after incubation with TNF‐α, Mir155, TR‐155 and tFNAs. (B) Flow cytometry analysis of NPCs proliferation after incubation with TNF‐α, Mir155, TR‐155 and tFNAs. (C) Immunofluorescence image of LC3b expression in NPCs and the statistical analysis of the fluorescence intensity (LC3b: red, nucleus: blue). (D) Immunofluorescence image of P26 expression in NPCs and the statistical analysis of the fluorescence intensity (P26: red, nucleus: blue). (E) Immunofluorescence image of Col II expression in NPCs and the statistical analysis of the fluorescence intensity (Col II: red, nucleus: blue). (F) Immunofluorescence image of BMP‐2 expression in NPCs and the statistical analysis of the fluorescence intensity (BMP‐2: red, nucleus: blue). (G) Gene expression level of LC3b, P26, Col II and BMP‐2. Data represented the mean ± SD. Statistical analysis was performed with one‐way ANOVA. (*) *p* < 0.05; (**) *p* < 0.01; (***) *p* < 0.001; (****) *p* < 0.0001. NPC, nucleus pulposus cell; tFNA, tetrahedral framework nucleic acid.

Excessive apoptosis of NPCs is closely linked to IDD.^27^ Decline of active cells within IVD leads to a diminished synthesis of ECM, forming the pathological basis of IDD.^22^ TNF‐α plays a critical role in the inflammation and apoptosis processes of the NPCs. Previous studies have indicated that a concentration of 100 ng/mL of TNF‐α can induce an increase in NPCs apoptosis.^28^ In this experiment, we employed flow cytometry to detect cell apoptosis and found that TNF‐α treatment significantly increased the percentage of apoptotic cells. Additionally, the introduction of miR155 after TNF‐α treatment did not reduce NPC apoptosis. However, introducing tFNAs or TR155 post TNF‐α treatment reversed TNF‐α‐induced apoptosis in NPCs, and the difference was statistically significant (*p* < 0.001) (Figure 2A). Simultaneously, we conducted live/dead staining to assess cell apoptosis and reached similar results (Figure 2B). These results suggested that TR155 could inhibit TNF‐α‐induced apoptosis of NPCs.

ECM plays a crucial role in sustaining the physiological function of IVD. Collagen II (Col II), a key component of ECM, contributes to maintaining IVD elasticity and osmotic pressure, with a significant correlation between the reduction of collagen II and IDD.^29^ In this study, NPCs were treated with TNF‐α first and then intervened with miR155, tFNAs or TR155. Immunofluorescence staining showed reduced collagen II after TNF‐α intervention (*p*<0.01). miR155 had no notable effect, but tFNAs or TR155 reversed this decline. Significantly, TR155 improved TNF‐α‐induced Col II reduction (*p*<0.001) (Figure 2C). We then utilised qPCR analysis to evaluate the expression of Col II at mRNA level, and the outcomes revealed a noteworthy reversal and enhancement in the expression of the Col II with the introduction of TR155 (Figure 2G). Prior studies have demonstrated a positive relationship between BMP‐2 and the expression of Col II in NP.^30^ Hence, we subsequently investigated the expression of BMP‐2, a kind of protein that is closely related with the osteogenic process. Immunofluorescence revealed TNF‐α suppressed BMP‐2 expression (*p* < 0.05). miR155 had no pronounced effect, while tFNAs or TR155 could reverse the decreasing tendency. Notably, TR155 significantly improved TNF‐α‐mediated BMP‐2 decline (*p* < 0.001) (Figure 2D). The qPCR results further suggested that TR155 counteracted the TNF‐α‐induced inhibition of BMP‐2 expression (*p*<0.01) (Figure 2G).

LC3B induces cell apoptosis by activating the extrinsic apoptotic pathway through dynamic interaction with Fas protein.^23^ p62, with multiple functions, is also actively involved in regulating biological processes like cell proliferation, differentiation, autophagy and apoptosis.^24^, ^25^ The increment of LC3B expression together with the decline in p62 expression would result in excessive cell apoptosis. Immunofluorescence staining results indicated an increased LC3b expression (*p* < 0.001) and a decreased p62 (*p* < 0.001) expression after TNF‐α induction. On the other hand, tFNAs or TR155 interventions both reversed the above trends (Figure 2E,F). The corresponding qPCR analysis confirmed a consistent result regarding LC3b and p62 expression levels (Figure 2G). In conclusion, TR155 was proved to inhibit the apoptosis of NPCs induced by TNF‐α, and the protective effect was more pronounced compared to miR155 or tFNAs, respectively.

### 3.3 TR155 improved the mitochondrial function of NPCs

**FIGURE 3:**
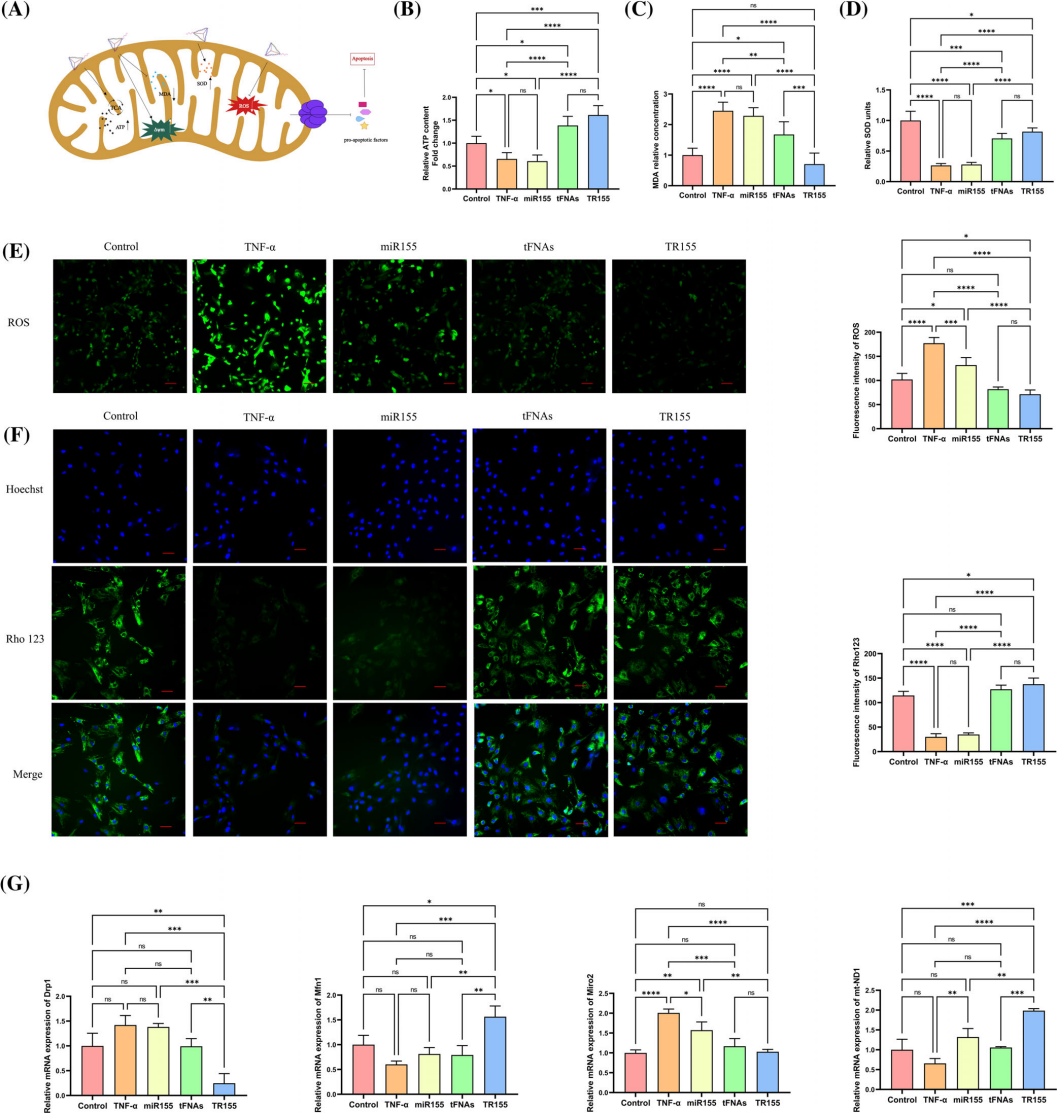
TR155 ameliorated the impact of TNF‐α on the mitochondrial function of NPCs. (A) Brief description the effect of TR155 on mitochondria. (B) The detection of mitochondrial ATP production in five experimental groups. (C) The detection of mitochondrial MDA production in five experimental groups. (D) The detection of mitochondrial SOD production in five experimental groups. (E) Immunofluorescence measurement and qualitative analysis of ROS expression in NPCs. (F) Immunofluorescence images and qualitative analysis of mitochondrial membrane potentials (Rhodamine 123 Assay). (G) Gene expression level of Drp1, Mfn1, Miro2 and mt‐ND1. Data represented the mean ± SD, *n* = 3. Statistical analysis was performed with one‐way ANOVA. (*) *p* < 0.05; (**) *p* < 0.01; (***) *p* < 0.001; (****) *p* < 0.0001. MDA, malondialdehyde; NPC, nucleus pulposus cell; ROS, reactive oxygen species; SOD, superoxide dismutase; tFNA, tetrahedral framework nucleic acid.

Previous research has identified that mitochondrial pathway plays a significant role in the apoptosis process of NPCs.^8^ Mitochondria are pivotal cell organelles for ATP generation, the primary energy source for all kinds of cellular activities. Hence, ATP production level is regarded as a major indicator for mitochondrial function. Our study first explored the ATP level in NPCs to understand the direct cellular response to TNF‐α, miR155, tFNAs, and TR155 (Figure 3A). After the introduction of TNF‐α to NPCs, ATP production significantly decreased (*p* < 0.05), yet miR155 treatment had no notable impact on ATP level. In contrast, tFNAs or TR155 treatment both significantly increased ATP production (*p* < 0.001), with TR155 showing the most pronounced impact (Figure 3B). Subsequently, we examined the production levels of two oxidative stress indicators, specifically MDA and SOD. As previously noted, SOD and MDA exert contrasting impacts on cell survival. SOD functions protectively, whereas MDA operates inversely, exerting detrimental effects.^31^ After TNF‐α induction on NPCs, MDA production notably increased (*p* < 0.001), while SOD production significantly decreased (*p* < 0.001). MiR155 treatment didn't notably change these markers, yet tFNAs or TR155 reversed the tendency in MDA (Figure 3C) and SOD (Figure 3D) levels, with TR155 showing the most significant change.

Excessive ROS production represents mitochondrial dysfunction and early apoptosis,^32^ contributing to IDD disease progression. Therefore, suppression of ROS‐mediated processes is crucial in averting IDD by preventing and mitigating cell apoptosis. As shown in Figure 3E, TNF‐α could significantly increase the generation of ROS in NPCs, while tFNAs or TR155 managed to reverse TNF‐α‐induced ROS production with statistically significant differences (*p* < 0.001). Specifically, TR155 exhibited a greater reduction in ROS level compared to tFNAs. The above outcomes indicated that TR155 had an inhibitory effect on the TNF‐α‐induced ROS generation in NPCs.

In order to further explore the mechanism of mitochondrial dysfunction in NPCs, we conducted additional studies on the MMP. When mitochondria become damaged under various circumstances, a decrease in MMP is often observed.^33^ After TNF‐α intervention, MMP significantly decreased (*p* < 0.001). Following the addition of miR155, there was no significant improvement in MMP level. However, in the groups where tFNAs or TR155 were introduced, the declining trend of MMP was reversed. In the TR155 group, MMP level was even higher than that of the control group (*p* < 0.05) (Figure 3F).

Additionally, we evaluated the mRNA expression of key mitochondrial dynamic genes. Mitochondrial function hinges on the balance between fusion and fission, where fusion aids ATP synthesis, new mitochondria formation, and damaged mitochondria repair. Increased fission, however, impairs mitochondrial function.^34^ The results of our study demonstrated that after TNF‐α induction, mitochondrial fission increased with elevated Drp1 and reduced Mfn1 expression. Notably, TR155 reversed this trend and promoted mitochondrial fusion by downregulating Drp1 and upregulating Mfn1 expression (Figure 3G). Simultaneously, TR155 treatment also reversed the changes in mRNA expression of mitochondrial Rho GTPase 2 (Miro2) and mitochondrial DNA (mt‐ND1) induced by TNF‐α (Figure 3G). The above results demonstrated that TR155 could assist in maintaining mitochondrial dynamics to alleviate the mitochondrial dysfunction induced by TNF‐α.

### 3.4 TR155 mitigated NPC apoptosis by modulating the Bcl‐2/Bax pathway

**FIGURE 4:**
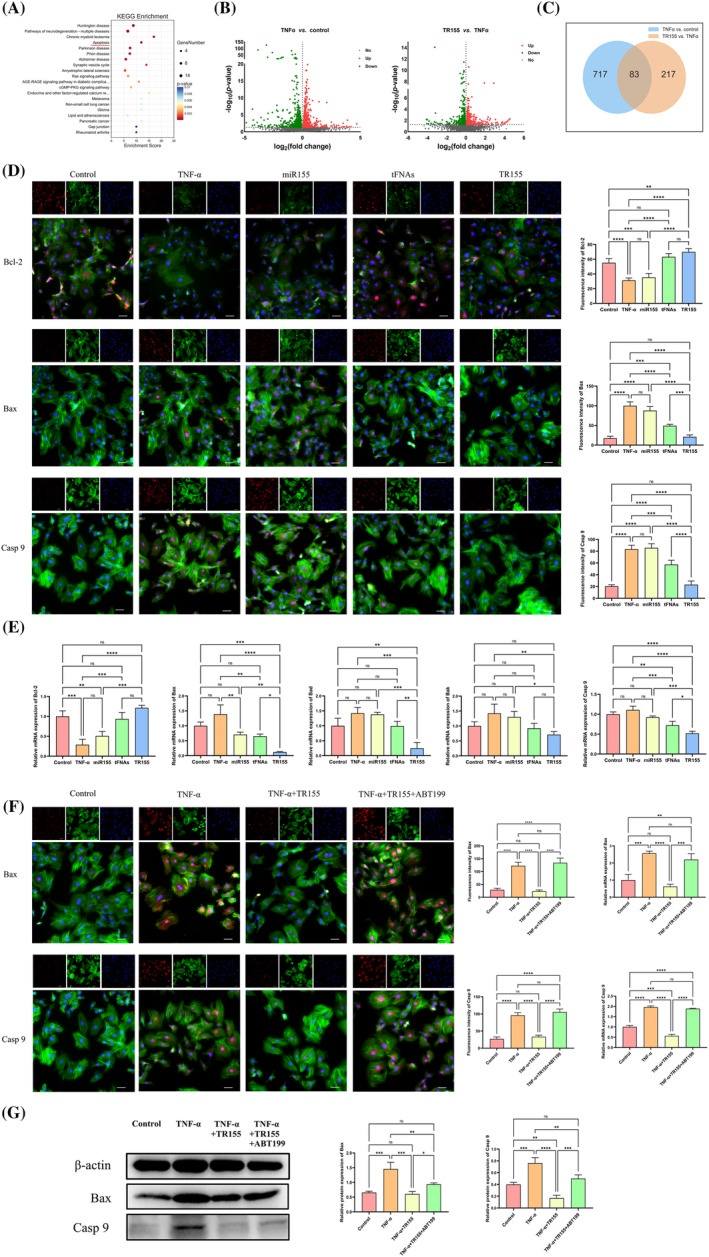
Effects of TR155 on the transcriptome of apoptosis in NPCs. (A) The result of KEGG enrichment analysation. (B) Volcano plots analysis of up and down‐regulated genes after TNF‐α treatment and tFNAs treatment, indicating apoptosis related genes (genes exhibiting a fold change in expression greater than 2 or less than 0.5, *p*<0.05). (C) Venn diagrams of down‐regulated genes after TNF‐α treatment and up‐regulated genes after TR155 treatment. (D) Immunofluorescence measurement and qualitative analysis of Blc‐2, Bax and Cas9 expression in five experimental groups. (E) Gene expression level of Blc‐2, Bax, Bad, Bak and Caspase9. (F) Immunofluorescence measurement, quantitative analysis and gene expression level of Bax and Caspase9 in four experimental groups. (G) Protein expression of Bax and Caspase9 by Western blot. Data represented the mean ± SD, *n* = 3. Statistical analysis was performed with one‐way ANOVA. (*) *p* < 0.05; (**) *p* < 0.01; (***) *p* < 0.001; (****) *p* < 0.0001. NPC, nucleus pulposus cell; tFNA, tetrahedral framework nucleic acid.

Based on the results that indicated the anti‐apoptotic and mitochondrial protective effects of TR155, we hypothesised that it acted on the mitochondria‐mediated apoptosis pathway. Further exploration through high‐throughput transcriptome sequencing (RNA‐seq) unveiled significant changes in 217 protein‐coding genes in the TR155‐treated group compared to the TNF‐α group (Figure 4C). The genes upregulated or downregulated following TNF‐α induction were individually intersected with the genes downregulated or upregulated after TR155 treatment, leading to a total of 83 identified genes (Figure 4C). KEGG analysis revealed that some of these genes were closely associated with cellular apoptosis (Figure 4A). During the analytic process, genes were sorted as upregulated or downregulated with fold changes >2 or <0.5 and a *p*‐value <0.05 (Figure 4B). Ultimately, we identified the Bax gene as the target, which is believed to be intricately linked to cell apoptosis. Besides Bax, we also examined its counterpart, Bcl‐2. Immunofluorescence staining revealed that TNF‐α decreased Bcl‐2 expression but increased the expression of Bax and Caspase9 in NPCs. Either TR155 or tFNAs could reverse this tendency, with TR155 showing the most significant reversal (Figure 4D). Additionally, qPCR was used to evaluate the expression of the anti‐apoptotic gene Bcl‐2 and pro‐apoptotic genes Bax, Bad, Bak and Caspase9. TNF‐α decreased Bcl‐2 expression, while boosting the expression of Bax, Bad, Bak and Caspase9. TR155, on the contrary, significantly altered this gene expression pattern towards the opposite direction (Figure 4E).

To support our hypothesis, we used ABT‐199, a potent Bcl‐2 inhibitor in the in‐vitro experiment that mimicked TNF‐α‐induced NPC apoptosis. Immunofluorescence staining results showed increased expression of Bax and Caspase9 after TNF‐α, which was reversed by TR155 treatment. However, the combination of TR155 and ABT199 still increased Bax and Caspase9 expression (Figure 4F). To validate our speculation comprehensively, we then measured mRNA expression in all groups and observed consistent trends. TNF‐α decreased Bcl‐2 expression but increased the expression of Bax, Bad and Caspase9 at mRNA level. TR155 reversed these changes, but the effect vanished when TR155 and ABT199 were added simultaneously (Figure 4F). Western Blot results demonstrated that TR155 treatment significantly inhibited the expression of Bax and Caspase9. However, this inhibitory effect is nullified upon the addition of ABT199 (Figure 4G). These findings strongly suggested that TR155 could restore the balance between pro‐apoptotic Bax and anti‐apoptotic Bcl‐2 at both protein and mRNA level.

### 3.5 TR155 alleviated puncture‐induced rat IDD in vivo

**FIGURE 5:**
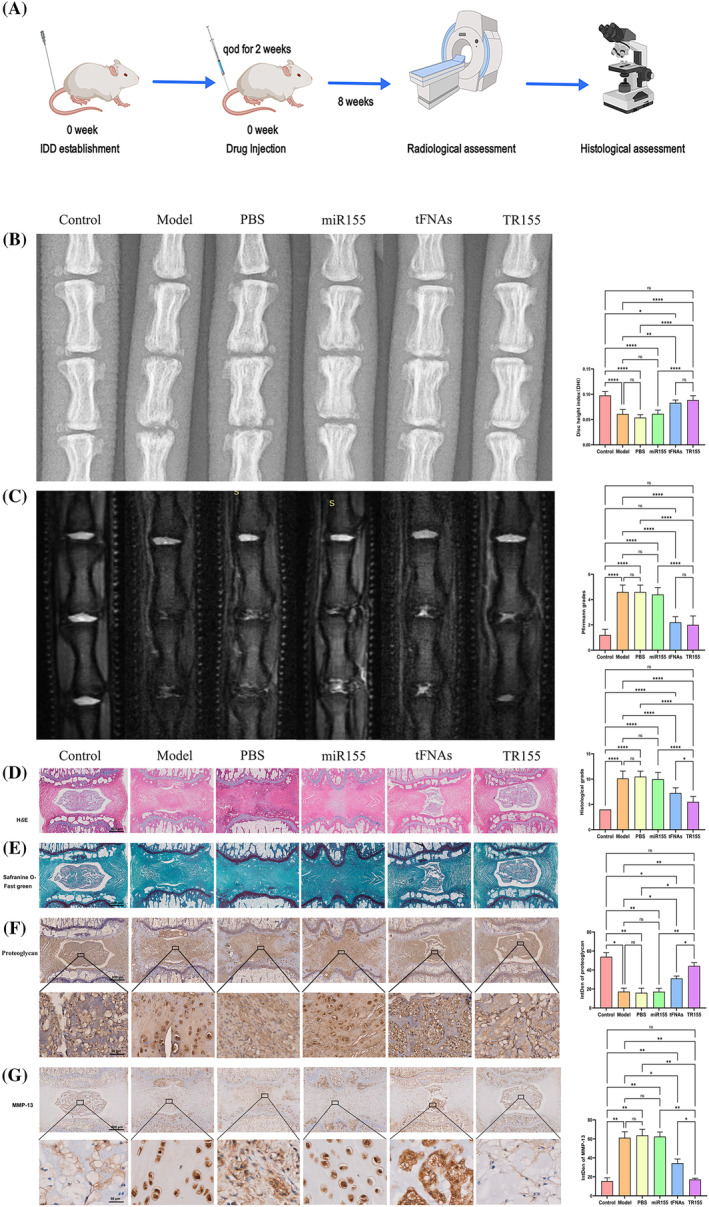
TR155 alleviated puncture‐induced rat IDD in vivo. (A) Brief description of the in vivo experiment process. (B) MRI Imaging of the intervertebral discs. (C) X‐ray images of the intervertebral discs. (D) Histological images of haematoxylin and eosin (H&E) staining of the intervertebral disc tissue, scale bar = 800 μm. (E) Safranin‐O/Fast staining of the intervertebral disc tissue, scale bar = 400 μm. (F) Immunohistochemical staining of proteoglycan, scale bar = 800 μm. (G) Immunohistochemical staining for MMP‐13, scale bar = 800 μm. (H) Disc height index (DHI) to evaluate IDD disease status. (I) Pfirrmann grading system to classify the intervertebral discs. (J) Histological assessment of intervertebral discs. (K) Quantitative analysis of proteoglycan expression. (L) Quantitative analysis of MMP‐13 expression. Data represented the mean ± SD, *n* = 3. Statistical analysis was performed with one‐way ANOVA. (*) *p* < 0.05; (**) *p* < 0.01; (***) *p* < 0.001; (****) *p* < 0.0001. IDD, intervertebral disc degeneration.

To assess the potential of TR155 in mitigating IDD, we established an IDD model in SD rats via needle puncture. Drugs were administered every other day using a 33G needle based on the assigned groups (Figure 5A). Variations in both the DHI and magnetic resonance imaging (MRI) signal intensity were documented. As shown in Figure 5B,H, x‐ray images indicated that, compared to the sham group with IDD gap collapse and endplate cartilage destruction after puncture, the DHI in the PBS and miR155 groups did not show significant improvement. In contrast, the tFNAs and TR155 groups showed slight improvement in DHI, although it still remained lower than the control group. Changes in IVD signal on T2‐weighted MRI images are closely correlated with the degree of degeneration, with lower signal intensity indicating more severe disc degeneration. As depicted in Figure 5C, after an 8‐week treatment period, the IVD in the sham group, PBS group, and miR155 group all demonstrated a low signal alteration. Conversely, the tFNAs group exhibited a high signal change in the NP region. Additionally, the TR155 group displayed an even larger area of high signal in the NP region compared to the tFNAs group. According to the Pfirrmann grading method for IVD classification, the above findings indicated higher grades in the sham, PBS, and miR155 groups. In contrast, the tFNAs and TR155 groups showed significantly lower grades, with the grade of TR155 group approaching the grade of the control group (Figure 5I). The uniformity in DHI and MRI modifications across the groups suggested a promising prospect for the effectiveness of TR155 in the treatment of IDD.

In comparison to the sham group, as evidenced by H&E and safranin O‐fast green staining, the TR155 group exhibited a notable inclination towards improved IDD (Figure 5D,E). H&E staining demonstrated a well‐defined boundary between NP and AF in the TR155 group, with NP morphology similar to that of the control group. Conversely, the tFNAs group exhibited a reduced NP area with an unclear boundary, while NP in the other three groups was replaced by fibrous tissue (Figure 5D). Safranin O‐Fast Green staining was employed for evaluating the presence of proteoglycan (orange) and collagen (green) within NP. As depicted in Figure 5E, staining in the TR155 group to a great extent resembled that of the control group, demonstrating a notable abundance of proteoglycans within the NP region. In contrast, in the other four groups, there was always replacement of proteoglycans by collagen to various degree and the variation was corroborated by the histological scores (Figure 5J).

To validate the above findings, proteoglycan and MMP13 expressions at mRNA level were as well examined across all experimental groups. The TR155 group demonstrated a significant upregulation of proteoglycans, resembling the levels in the control group (Figure 5F,K). Conversely, a distinct decreasing trend in MMP‐13 expression was evident in the TR155 group when contrasted with the sham group (Figure 5G,L). The above in‐vivo results indicated that TR155 could help maintain the balance in ECM metabolism to protect the normal NP morphology and delay the progression of IDD.

## 4 DISCUSSION

Excessive apoptosis of NPCs plays an important role in the disease progression of intervertebral disc degeneration.^5^ Our experimental results showed that TR155, the complex formed by tetrahedral framework nucleic acid carrying microRNA‐155, demonstrated a significant effect on the proliferation of NPCs and could alleviate cell inflammation and apoptosis induced by TNF‐α. The results also showed that TR155 significantly increased the expression level of type 2 collagen in vivo, and the expression of BMP‐2 protein, which was positively correlated with the expression of type 2 collagen, increased significantly as well. At the same time, we also found that the expression level of LC3b and P62, two markers that are closely related to autophagy and apoptosis, were significantly altered. The above evidence confirmed that TR155 could help maintain the homeostasis of the cellular microenvironment in the intervertebral disc.

The mitochondria mediated apoptotic pathway is recognised as one of the key mechanisms in cell apoptosis. In this study, we evaluated the effect of TR155 on mitochondrial function by detecting intracellular ROS and ATP production levels, as well as the production levels of oxidative stress markers SOD and MDA in mitochondria. We found that both TR155 and tFNAs increased ATP production and enhanced mitochondrial activity; meanwhile the increase in MDA production and the decrease in SOD production were also observed. SOD is believed to play a protective role in the survival of NPCs, while MAD has a reverse effect. Besides, TR155 and tFNAs both had a significant inhibitory effect on TNF‐ α induced ROS generation. The above results demonstrated the positive promoting effect of TR155 and tFNAs on mitochondrial function, and between them, the effect of TR155 is significantly superior to tFNAs.

The balance between mitochondrial fission and fusion is crucial for maintaining mitochondrial homeostasis.^35^, ^36^, ^37^ Upregulation of DRP1 expression and inhibition of Mfn1 expression can lead to excessive mitochondrial division. We found that TR155 could promote mitochondrial fusion and inhibit mitochondrial fission by regulating the expression of DRP1 and Mfn1. Furthermore, it contributed to the synthesis of ATP, the formation of new mitochondria, and the repair of damaged mitochondria. The above results indicated that TR155 played a pivotal role in maintaining mitochondrial homeostasis and could therefore alleviate TNF‐ α induced mitochondrial dysfunction, and the above effects were all superior when compared to pure tFNAs and microRNA155.

Through transcriptome sequencing analysis, two genes closely related to cell apoptosis known as Bax and Bcl‐2 were spotted. The Bax and Bcl‐2 family were found to be crucial in maintaining the balance between pro‐apoptotic and anti‐apoptotic effects, and TR155 altered TNF‐ α induced apoptosis by increasing the expression of Bax while hindering the expression of Bcl‐2. However, this effect was reversed after the use of Bcl‐2 pathway inhibitor ABT‐199. The above evidence illustrated that TR155 could alleviate TNF‐ α induced apoptosis of NPCs by regulating the Bcl‐2/Bax pathway. We as well evaluated the potential of TR155 in alleviating IDD by DHI and MRI imaging in a rat IDD model, and the corresponding results were consistent with our findings from previous in‐vitro experiments, indicating that TR155 had promising application prospects for treating IDD in future.

## 5 CONCLUSIONS

In summary, we have successfully developed a complex named TR155 using tetrahedral framework nucleic acids for loading miRNA155. This complex could help treating intervertebral disc degeneration in vitro by suppressing excessive cell apoptosis and extracellular matrix degradation. In vitro, TR155 could intervene with the apoptotic process of NPCs by regulating the mitochondria‐mediated Bcl‐2/Bax pathway. More research on improving the loading efficiency and targeting capacity of tFNAs is needed to fully unleash the therapeutic potential of nucleic acid nanomedicine in the treatment of intervertebral disc degeneration.

## AUTHOR CONTRIBUTIONS

Zhuhai Li and Yuanlin Tang conceived and planned the experiments. Zhuhai Li, Yuanlin Tang, Lihang Wang and Kai Wang carried out the experiments. Zhuhai Li and Yuanlin Tang contributed to the interpretation of the results. Yuanlin Tang took the lead in writing the manuscript. Shishu Huang and the corresponding author Yu Chen provided consultation for the final version of the manuscript. All authors provided critical feedback and helped shape the research, analysis and manuscript.

## FUNDING INFORMATION

This work was supported by Natural Science Foundation of Sichuan Province (2022NSFSC0367), Natural Science Foundation of Guangxi Province (2024GXNSFBA010270), the Science and Technology Department of Sichuan Province (2019YFQ0003 and 2022YFS0051).

## CONFLICT OF INTEREST STATEMENT

The authors declare no conflicts of interest.

## Data Availability

The data that support the findings of this study are available from the corresponding author upon reasonable request.
